# Epilepsy

**Published:** 1891-04-11

**Authors:** 


### THE PRACTITIONER'S MIRROR AND RETROSPECT.

[*The Editor will be glad to receive offers of co-operation and contributions from members of the profession. All letters should be addressed to* THE EDITOR, THE LODGE, PORCHESTER SQUARE, LONDON, W.]

### MEDICINE.

### EPILEPSY.

Comparative results of the various remedies employed in the treatment of epilepsy will always be most fallacious until it is realised that distinctions must be drawn between the different classes of cases. It has been usual to divide cases into those of "grand mal" and "petit mal," and though for clinical purposes such distinctions are of value for the sake of symptomatic differentiation, from a therapeutical aspect this classification is lamentably insufficient. Of late epilepsy resulting from a more or less localised cortical lesion, and bearing distinctive features, have been differentiated as Jacksonian epilepsy. There are, however, a large number of cases, which may be regarded as idiopathic epilepsy, originating not in any cortical or peripheral irritation, but in a degenerate condition of the higher nerve centres, commonly occurring in persons with a well-marked family history of insanity, and in those born of drunken parents. In these cases we can often do something towards warding off the recurrence of epileptic fits for a time, but cures must be few and far between, and the indiscriminate inclusion of this class of epileptic with that class in which the epileptic attacks depend or, at any rate, originated in some peripheral lesion, must render statistics of results more or less misleading.

Dr. Stevens, of New York, has directed attention in his work on "Functional Nervous Diseases," to his conclusions based on the results of the work of a number of years upon the nervous disorders resulting from anomalies of the eye-muscles. He believes that they are the frequent cause of epilepsy, chorea, and numerous other functional diseases. Certainly his results from treating these affections by tenotomies and spectacles seem very convincing for improvement in most of the cases of epilepsy, and cures in many are claimed by him to result from his treatment, which consists chiefly in repeated partial tenotomies.

Dr. M. Allen Starr has investigated the relation of this form of peripheral irritation which it has been stated induces epilepsy, and we do not think he has under-estimated the influence of eye-strain as a factor in causing epilepsy. In his paper, after referring to the neuroses which have from time to time been claimed to result from irritation of the genital organs, the nasal and aural cavities, and even the teeth, he went on to discuss the want of balance of the various ocular muscles in producing epilepsy. He believes that though each form of peripheral irritation may produce nervous symptoms, no one form has such a special importance as to warrant the extravagant statements made by their earliest supporters, and that while, no doubt, it was quite true that many nervous disorders are secondarily produced by local irritations, it is a fact that nervous affections thus produced bear very different characteristics from cases of true epilepsy, and the well-known forms of mental disease. When peripheral irritation exists sufficient in degree to be injurious, nature points this out by producing a sense of discomfort in the part irritated, and it was Dr. Starr's experience that unless an extreme degree of discomfort exists, it is really a matter of comparatively little importance. He could only cite one case of true nervous manifestation caused by eye-strain, out of three thousand cases of nervous disease, and he concluded that while eye-strain or other peripheral irritation may be a source of nervous manifestation, it is a rare cause of nervous disease, and he does not believe that true epilepsy or chorea can be produced by eye-strain or cured by its relief, and is of opinion that the general indiscriminate recommendation of treatment directed to the relief of supposed eye-strain in these diseases, will soon come to be recognised as malpractice. These valuable observations are supported by Dr. St. John Roosa, who, out of three thousand five hundred and eighty-four cases showing errors of refraction, did not find a single case of epilepsy or hysteria, and only three were choreic cases.

While, however, these authorities have so completely refuted the extravagant views that have been propounded in relation to eye-strain as a cause of epilepsy, it must not be forgotten that peripheral lesions do cause epilepsy, and a good instance of this is reported by Dr. Crossfield. His patient, a young man, had had epileptic attacks about twice a month for six years. During the whole of this long period he took bromides in various forms, with cod-liver oil and hypophosphites. Observing that the patient was a mouth-breather, Dr. Crossfield examined the nares, and discovered a marked hypertrophy of the mucous membrane of the inferior turbinated bone, causing almost complete obstruction. There was also a mass of hypertrophied adenoid tissue of the naso-pharynx and elongation of the uvula with hypertrophy. All these abnormalities were removed, and though it is now three years since the operations were completed, he has had only one epileptic seizure since the commencement of the treatment.

With regard to the medicinal treatment of epilepsy, M. Cornet has published the results of his experiments which he made under the direction of M. Bourneville as to the action of bromide of gold, bromide of camphor, and picrotoxine in this affection. He found that while bromide of gold has a certain favourable influence in some cases, it is inferior in its action to bromide of potassium. Our own experience of the bromide of gold entirely accords with this view, for although one-fortieth of a grain to one-tenth of a grain will often control the disease and prevent the recurrence of the attacks, the patients complain that they do not feel so well, and often beg to be put on bromide of potassium again. M. Cornet administered the bromide of gold in doses as large as half-a-grain without producing any unfavourable result. The bromine is slowly eliminated by the urine, and the gold accumulates in the liver, and only after a long time appears in the urine.

Bromide of camphor has a beneficial action upon the attacks of vertigo in epileptics, the attacks being less severe and less frequently recurring, or even disappearing altogether. Bromide of camphor is entirely eliminated by the kidneys, the bromine, as bromide of sodium, the camphor undergoing decomposition, and being eliminated indirectly. Picrotoxine in doses of one-fortieth to one-thirty-second of a grain, while not without beneficial action, did not appear to give very good results.

Biborate of soda has been advocated, especially as a substitute for bromides in nocturnal epilepsy. Dr. Stewart reported seven cases in which he used borax. The first case was in a girl, aged thirteen, in whom the attacks dated from birth, and varied in frequency from two to twelve in the twenty-four hours, the fits generally occurring at night. The fits gradually diminished in frequency under the administration of borax, and in nine weeks they ceased entirely. His second case was an epileptic of five years' standing, whose fits averaged a hundred and one monthly. The fits very rapidly diminished in frequency till in the fifth month not a single fit, and in the sixth only one occurred. In two other cases similar results were obtained. In the remaining three, the fits were both nocturnal and diurnal, and while the nocturnal seizures were controlled by the borax, no effect on the diurnal fits was observed. Drs. Russell and Taylor, after treating twenty cases with borax, in doses of from seven to sixty grains daily, concluded that it was beneficial in its action, but far inferior to the bromides.

Of all the numerous sedative drugs which have been advocated in the treatment of epilepsy, none come up to the bromides, especially bromide of potassium which still maintains its position. While probably it is desirable to begin the treatment of all cases of epilepsy in which the attacks are at all frequent with bromide of potassium, there is much to be said for the tonic treatment if cure and not palliation is to be hoped for. Dr. Thomson advises the administration of cod-liver oil and phosphorus, and the total exclusion of butchers' meat for at least two years. The persistence in treatment over a long period is certainly most essential.

Small doses of strychnine have been given with good results, but such drugs must be administered with the greatest caution.

